# Die Medizininformatik-Initiative im Überblick – Aufbau einer Gesundheitsforschungsdateninfrastruktur in Deutschland

**DOI:** 10.1007/s00103-024-03887-5

**Published:** 2024-06-05

**Authors:** Sebastian C. Semler, Martin Boeker, Roland Eils, Dagmar Krefting, Markus Loeffler, Jens Bussmann, Frank Wissing, Hans-Ulrich Prokosch

**Affiliations:** 1 Koordinationsstelle der Medizininformatik-Initiative (MII), TMF – Technologie- und Methodenplattform für die vernetzte medizinische Forschung e. V., Berlin, Charlottenstraße 42, 10117 Berlin, Deutschland; 2 Institut für Künstliche Intelligenz und Informatik in der Medizin, Lehrstuhl für Medizinische Informatik, Klinikum rechts der Isar, School of Medicine and Health, Technische Universität München, München, Deutschland; 3 Health Data Science Unit, Medizinische Fakultät Heidelberg, Universität Heidelberg, Heidelberg, Deutschland; 4 Institut für Medizinische Informatik, Universitätsmedizin Göttingen, Göttingen, Deutschland; 5 Institut für Medizinische Informatik, Statistik und Epidemiologie, Universität Leipzig, Leipzig, Deutschland; 6 VUD Verband der Universitätsklinika Deutschlands e. V., Berlin, Deutschland; 7 MFT Medizinischer Fakultätentag der Bundesrepublik Deutschland e. V., Berlin, Deutschland; 8 Lehrstuhl für Medizinische Informatik, Friedrich-Alexander-Universität Erlangen-Nürnberg, Erlangen, Deutschland

**Keywords:** Sekundärnutzung von Gesundheitsdaten, Forschungsdateninfrastruktur, Datennutzung und Datenzugang, Medizininformatik, Standardisierung, Secondary use of health data, Research data infrastructure, Data use and access, Medical informatics, Standardization

## Abstract

Die vom Bundesministerium für Bildung und Forschung (BMBF) 2016–2027 geförderte Medizininformatik-Initiative (MII) schafft erfolgreich Grundlagen für die datenbasierte Medizin in Deutschland. Zur Stärkung der Lehre, Aus- und Fortbildung im Bereich der Medizininformatik und zur Kompetenzverbesserung in den medizinischen Datenwissenschaften wurden im Rahmen dieser Förderung 51 neue Professuren, 21 wissenschaftliche Nachwuchsgruppen und verschiedene neue Studiengänge eingerichtet. Eine die gesamte Universitätsmedizin und ihre Partner umfassende gemeinsame dezentral föderierte Forschungsdateninfrastruktur wurde in Gestalt der Datenintegrationszentren (DIZ) an allen Standorten und dem Deutschen Forschungsdatenportal für Gesundheit (FDPG) als zentralem Zugangspunkt geschaffen. Für die Sekundärnutzung von Behandlungsdaten wurde ein modularer Kerndatensatz (KDS) definiert und unter konsequenter Nutzung internationaler Standards (z. B. FHIR, SNOMED CT, LOINC) implementiert. Als Rechtsgrundlage wurde eine behördlich genehmigte bundesweite breite Einwilligung (Broad Consent) eingeführt. Erste Datenausleitungen und Datennutzungsprojekte sind durchgeführt worden, eingebettet in eine übergeordnete Nutzungsordnung und standardisierte vertragliche Regelungen. Die Weiterentwicklung der MII-Gesundheitsforschungsdateninfrastrukturen im kooperativen Rahmen des Netzwerks Universitätsmedizin (NUM) bietet einen hervorragenden Ausgangspunkt für einen deutschen Beitrag zum kommenden Europäischen Gesundheitsdatenraum (EHDS), der Chancen für den Medizinforschungsstandort Deutschland eröffnet.

## Abstract (en)

The Medical Informatics Initiative (MII) funded by the Federal Ministry of Education and Research (BMBF) 2016–2027 is successfully laying the foundations for data-based medicine in Germany. As part of this funding, 51 new professorships, 21 junior research groups, and various new degree programs have been established to strengthen teaching, training, and continuing education in the field of medical informatics and to improve expertise in medical data sciences. A joint decentralized federated research data infrastructure encompassing the entire university medical center and its partners was created in the form of data integration centers (DIC) at all locations and the German Portal for Medical Research Data (FDPG) as a central access point. A modular core dataset (KDS) was defined and implemented for the secondary use of patient treatment data with consistent use of international standards (e.g., FHIR, SNOMED CT, and LOINC). An officially approved nationwide broad consent was introduced as the legal basis. The first data exports and data use projects have been carried out, embedded in an overarching usage policy and standardized contractual regulations. The further development of the MII health research data infrastructures within the cooperative framework of the Network of University Medicine (NUM) offers an excellent starting point for a German contribution to the upcoming European Health Data Space (EHDS), which opens opportunities for Germany as a medical research location.

## Einleitung – Motivation und Historie

Mit dem vom Bundesministerium für Bildung und Forschung (BMBF) am 16.11.2015 veröffentlichten „Förderkonzept Medizininformatik“ [1] und der damit verbundenen Förderausschreibung wurde der Notwendigkeit einer zunehmenden Digitalisierung im Gesundheitswesen und dem Potenzial Rechnung getragen, mit verstärkter elektronischer Datenverarbeitung in der medizinischen Forschung Diagnostik und Therapie zu verbessern. Die förderpolitischen Ziele des „Förderkonzepts Medizininformatik“ sind vielfältig [2]:

- die Chancen der Digitalisierung in der Medizin nutzen,
- durch die Entwicklung innovativer IT-Lösungen die Forschungsmöglichkeiten und die Patientenversorgung verbessern,
- den Austausch und die Nutzung von Daten über die Grenzen von Institutionen und Standorten hinweg unterstützen und voranbringen,
- insbesondere den Austausch von Daten zwischen der Krankenversorgung und der klinischen und biomedizinischen Forschung und die Nutzung der Daten intensivieren,
- durch einen verbesserten elektronischen Daten- und Wissensaustausch aktuelle Forschungsergebnisse schneller und besser im Versorgungsalltag verfügbar machen und
- die hierfür erforderliche Kompetenz steigern und die Lehre, Aus- und Fortbildung im Bereich der Medizininformatik stärken.

Um diese Ziele zu erreichen, wurde zur Bildung von wissenschaftlichen Konsortien aufgerufen, die anhand bestimmter Use Cases innovative IT-Lösungen konzipieren und interoperabel umsetzen sollten, sodass eine übergreifende gemeinsame Forschungsdateninfrastruktur entsteht.

**Abb. 1:**
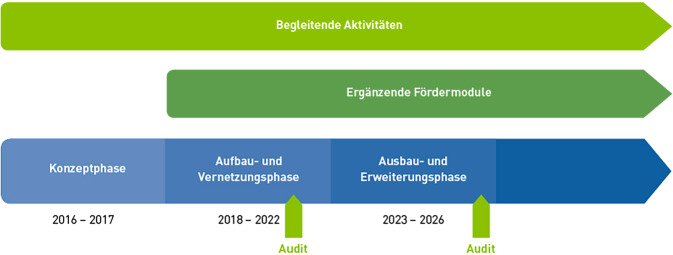
Förderphasen der Medizininformatik-Initiative des BMBF: Abgebildet sind die aufeinanderfolgenden Phasen der Förderung für die Konsortien sowie die parallel bzw. zeitversetzt geförderten weiteren Fördermodule (Use-Case-Projekte, Digitale FortschrittsHubs, Nachwuchsgruppen) und begleitende Aktivitäten (Koordinationsprojekt)

Das Förderprogramm sieht für die Medizininformatik-Initiative (MII) 3 Förderphasen vor (Abb. 1):

- Konzeptphase 2016–2017,
- Aufbau- und Vernetzungsphase 2018–2022,
- Ausbau- und Erweiterungsphase 2023–2026.

Gemäß Förderkonzept lag der Schwerpunkt hierbei zunächst auf den Standorten der Universitätsmedizin, welche ca. 1,8 Mio. Patientinnen und Patienten pro Jahr und damit 10 % aller stationären Fälle in Deutschland behandeln, mithin also für die MII einen substantiellen, für die Patientenversorgung relevanten Startpunkt darstellen. Im weiteren Verlauf wurden weitere Akteure des Gesundheitswesens einbezogen. Das Heben, Vernetzen und Nutzen von „Datenschätzen“ zugunsten der Patientenversorgung sind erklärtes Ziel der Initiative. Dies ist für Deutschland deshalb relevant, als sich die historisch gewachsene, vielfältige Fragmentierung der Zuständigkeiten und Datenbestände (Gesundheitsversorgung vs. Forschung, ambulanter vs. stationärer Sektor, regulatorische Kompetenzen auf Bundes- vs. Landesebene u. v. a.) als Hemmnis für eine wettbewerbsfähige Digitalisierung erwiesen hat. Mit dem Förderkonzept zur nationalen MII ergab sich eine gute Chance, die Medizininformatik an den deutschen Hochschulen zu stärken und auf nationalem Level den Einsatz moderner Informationstechnologie im Gesundheitswesen voranzubringen. Wichtige Impulse sollen für weite Bereiche des Gesundheitssystems und der Gesundheitswirtschaft – über Sektorengrenzen, Bundesländer und Einzelprojekte hinweg – gegeben werden, insbesondere zur Standardisierung und zur Interoperabilität als wichtigem Beitrag zur E‑Health-Entwicklung in Deutschland. Insbesondere hat sich die MII als ein prioritäres Ziel gesetzt, die breite Sekundärnutzung von Patientendaten für Forschungszwecke zu ermöglichen und somit die medizinische Forschung in Deutschland mit relevanten „Real-World-Daten“ zu stärken [3]. Über alle Phasen und Module des Förderrahmens wird die MII insgesamt mit rund 500 Mio. € Bundesmitteln gefördert.

## Vorgehensweise, Struktur und Governance

### Struktur und Governance

In der 9‑monatigen Konzeptphase wurden zunächst 7 Konsortien gefördert, um für die Aufbau- und Vernetzungsphase wissenschaftliche Konzepte auszuarbeiten. Auf Basis einer internationalen Begutachtung wurden daraus 4 Konsortien (DIFUTURE [4], HiGHmed [5], MIRACUM [6] und SMITH [7]) ausgewählt. Gleichzeitig waren aber alle Konsortien und die darin vertretenen Standorte der Universitätsmedizin verpflichtet, zusammen mit der parallel eingerichteten MII-Koordinationsstelle ein konsortienübergreifendes Konzept zu entwickeln, das eine deutschlandweite Nutzung von Daten aus der Krankenversorgung für die medizinische Forschung ermöglichen und die Initiative mit einer verbindlichen gemeinsamen Arbeitsplanung unterlegen sollte. Die MII-Koordinationsstelle wird betrieben von der TMF-Technologie- und Methodenplattform für die vernetzte medizinische Forschung e. V. (TMF), dem Medizinischen Fakultätentag der Bundesrepublik Deutschland e. V. (MFT) und dem Verband der Universitätsklinika Deutschlands e. V. (VUD).

**Abb. 2:**
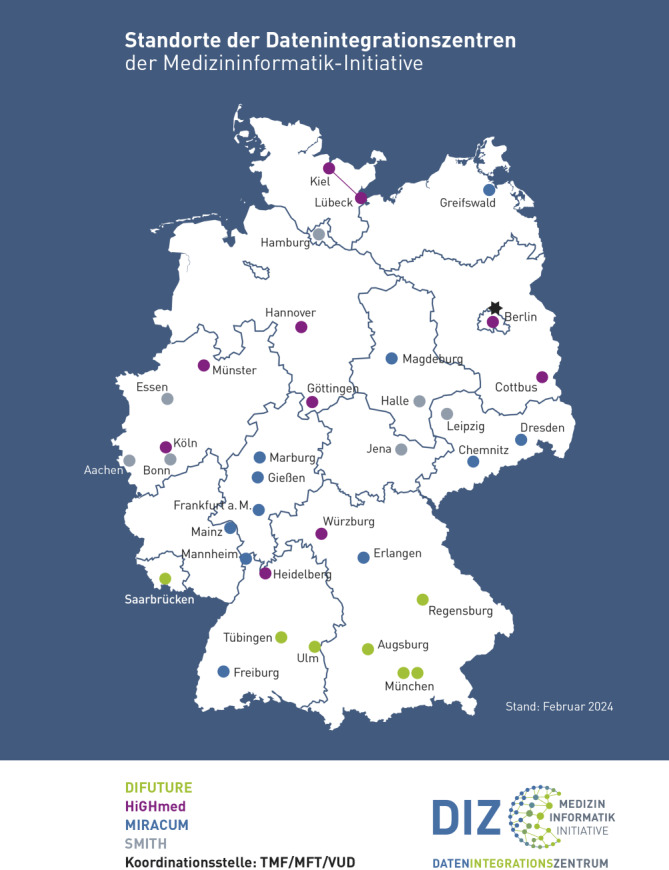
Standorte von Datenintegrationszentren (DIZ) der Medizininformatik-Initiative (MII) und deren Zugehörigkeit zu den MII-Konsortien (Stand: Anfang 2024). *Stern* Koordinationsstelle

Mit dem Start der ersten Hauptförderphase, der Aufbau- und Vernetzungsphase ab 2018, waren die Standorte und Partner der 3 nicht weiter geförderten Konsortien aus der Konzeptphase aufgefordert, sich möglichst zeitnah einem der geförderten Konsortien als neuer Projekt- oder Vernetzungspartner anzuschließen. Dieser Prozess konnte sehr früh abgeschlossen werden, sodass sich bereits 2019 alle deutschen Universitätskliniken einem der 4 Konsortien angeschlossen hatten und damit Teil der MII waren. An 29 Standorten wurde in der Aufbau- und Vernetzungsphase ein sogenanntes Datenintegrationszentrum (DIZ) etabliert, in welchem die Konzeptions- und Implementierungsarbeiten zur Umsetzung der jeweils eingereichten Konzepte durchgeführt wurden. Für die wenigen weiteren Universitätskliniken erfolgte der DIZ-Aufbau erst in der nachfolgenden Ausbau- und Erweiterungsphase ab 2023. Ebenso konnten sich ab 2023 erste nichtuniversitäre Standorte mit dem Aufbau eines DIZ der Initiative anschließen (Abb. 2).

### Nationales Steuerungsgremium

**Abb. 3:**
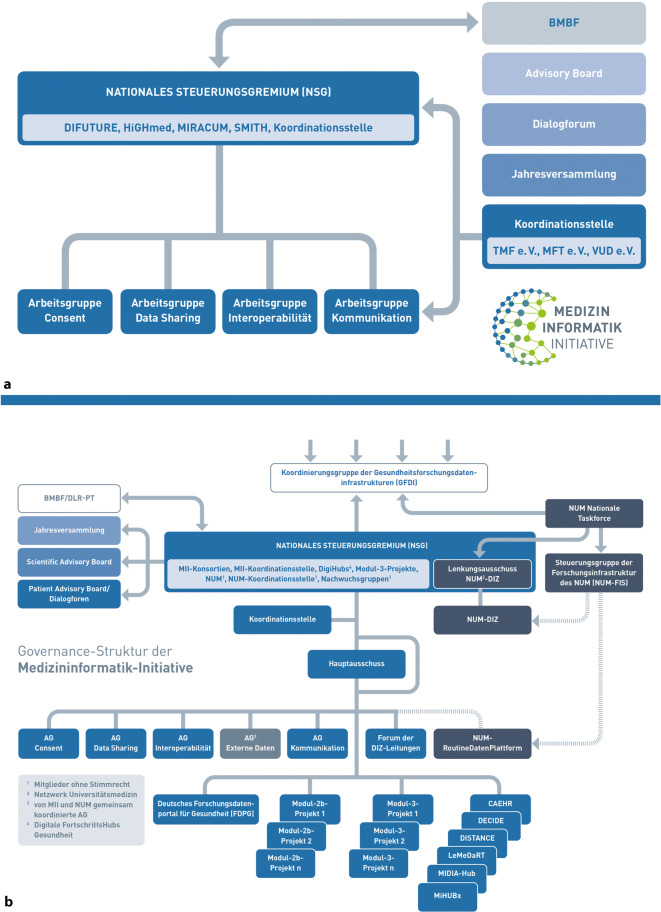
Governance der Medizininformatik-Initiative (MII): Das Nationale Steuerungsgremium (NSG) und die Arbeitsgremien der MII. **a** in der Aufbau- und Vernetzungsphase (2018–2022); **b** in der Ausbau- und Erweiterungsphase ab 2023 – komplexere Projektstruktur und erweiterte Repräsentanz der Projekte der unterschiedlichen Fördermodule. *Dunkel* dargestellt sind die Interaktions- und Kooperationspunkte mit dem Netzwerk Universitätsmedizin (NUM)

Um verbindliche Beschlüsse und Umsetzungen über Konsortien und Teilprojekte hinweg zu gewährleisten, wurde bereits zu Beginn der Konzeptphase 2016 das Nationale Steuerungsgremium (NSG) als oberstes Organ der MII eingesetzt, das Vertreterinnen und Vertreter der Konsortien gemeinsam mit der Vertretung der Koordinationsstelle bilden. Dabei setzt sich die Vertretung der Konsortien im NSG sowohl aus Expertinnen und Experten der Medizininformatik als auch aus Vertreterinnen und Vertretern von Vorständen der Universitätskliniken und der Dekanate zusammen, um die notwendige Umsetzung von Beschlüssen und Ergebnissen an den Standorten der Universitätsmedizin zu sichern. Das NSG wird in seiner Arbeit begleitet und unterstützt durch (a) die Koordinationsstelle, (b) das fördernde Ministerium (BMBF) und seinen Projektträger, (c) das Dialogforum, in welchem die Stakeholder eingebunden werden, um das Vorhaben über die reinen Forschungsfragestellungen hinaus zu justieren (Abb. 3a). Das NSG beruft einmal jährlich eine Jahresversammlung bzw. ein öffentliches Symposium sowie kontinuierlich zusammentretende Arbeitsgruppen (AGs) ein (die wiederum eigene Substrukturen einrichten können). Diese haben die Aufgabe, Entscheidungsvorlagen für die notwendigen Beschlüsse zu erarbeiten, die zur Sicherung der konsortienübergreifenden Kooperationsfähigkeit und zu einer internationalen Anschlussfähigkeit der aufzubauenden Datenintegrationsinfrastrukturen beitragen. An den AGs können sich alle Standorte der Universitätsmedizin in Deutschland mit ihren Expertinnen und Experten beteiligen. Punktuell werden hier auch externe Fachleute und Institutionen früh einbezogen (zum Beispiel Standardisierungsorganisationen, Fachgesellschaften und Fachgruppen). Um Doppelarbeiten zu vermeiden, werden bestehende Gremien und Arbeitsstrukturen insbesondere aus den 3 begleitenden Verbänden (TMF, MFT, VUD) einbezogen und für die MII genutzt (z. B. die AG Datenschutz bzw. die AG Biobanken der TMF).

Mit Beginn der Ausbau- und Erweiterungsphase 2023 wurde die Projektstruktur komplexer (Abb. 3b): Die Förderausschreibung für diese Phase sah verschiedene Fördermodule vor [8]. In Modul 1 werden die Konsortien als koordinative Strukturen der ihnen angeschlossenen Standorte gefördert. Modul 2 umfasst alle zentralen Strukturen – zum einen die Koordinationsstelle (Modul 2a), zum anderen Projekte, die zentrale Infrastrukturen und Dienstleistungen weiterentwickeln und dem gesamten Netzwerk anbieten (Modul 2b). Modul 3 umfasst sowohl methodische als auch klinische Use Cases, in denen als Leuchtturmprojekte zwingend konsortienübergreifend der Mehrwert der MII für die Gesundheitsversorgung und -forschung gezeigt werden soll. Diese Module werden nun über einen neuen Hauptausschuss gebündelt und finden im NSG ebenso Vertretung wie die bereits seit 2021 geförderten Digitalen FortschrittsHubs und die Nachwuchsgruppen (Abb. 3b). Auf diese Projekte der unterschiedlichen Fördermodule wird weiter unten näher eingegangen, ebenso auf die Verzahnung mit dem Netzwerk Universitätsmedizin (NUM).

### Arbeitsgruppen

Das NSG setzt für die erforderlichen fachlichen Arbeiten AGs ein, an denen Fachleute aller Konsortien, Module und Projekte der MII teilnehmen können. Die AGs erarbeiten und stimmen die Entscheidungsvorlagen für das NSG ab. Sie monitoren gemeinsam mit dem Hauptausschuss die Arbeiten der MII. Aktuell (Anfang 2024) gibt es 5 AGs zu folgenden Arbeitsbereichen: AG Consent, AG Data Sharing, AG Externe Daten, AG Interoperabilität, AG Kommunikation.

### Die Konsortien der MII

Über alle Phasen der MII wurden die folgenden 4 Konsortien gefördert, die neben der übergreifenden Zusammenarbeit in der MII auch eigene wissenschaftliche Entwicklungen verfolgen.

#### DIFUTURE – Data Integration for Future Medicine

DIFUTURE [4] umfasst Universitäten und Universitätskliniken an 7 Standorten: Augsburg, Homburg, München (Ludwig-Maximilians-Universität [LMU] und Technische Universität [TUM]), Regensburg, Tübingen und Ulm. DIFUTURE hat Schwerpunkte im Bereich Neurologie und Onkologie auf die Unterstützung von Frühdiagnose und Therapieentscheidungshilfen gesetzt. Insbesondere der Anwendungsfall multiple Sklerose zielt auf ein besseres Verständnis der Erkrankung und der krankheitsmodifizierenden Therapie ab. Dazu wurden Algorithmen entwickelt, um den Krankheitsverlauf bei Ausbruch vorherzusagen und frühzeitige personalisierte Behandlungsentscheidungen zu ermöglichen. Zum Ausbau der medizinischen Informatik wurden insgesamt 15 Professuren und 4 Nachwuchsgruppen an Standorten des DIFUTURE-Konsortiums eingerichtet. Darüber hinaus bietet DIFUTURE Bachelor- und Masterstudiengänge mit einem breiten Spektrum an Ausbildungsmöglichkeiten in der medizinischen Informatik an. Zu den angebotenen Studiengängen gehören „Biomedical Computing“, „Medical Information Sciences“ und „Medical Informatics“. Seit 2023 beteiligt sich DIFUTURE an den gemeinsamen MIRACUM-DIFUTURE-Kolloquien und -Schools.

#### HiGHmed

Das Konsortium HiGHmed [5] vereint die Expertise von 12 Universitätskliniken und medizinischen Fakultäten an den Standorten Berlin, Bielefeld, Cottbus, Göttingen, Hannover, Heidelberg, Kiel, Köln, Lübeck, Münster, Oldenburg und Würzburg. Im Jahr 2024 haben sich ein nichtuniversitärer sowie ein internationaler Standort dem Verbund angeschlossen (Robert-Bosch-Krankenhaus Stuttgart, Luxembourg Institute of Health). Alle Standorte etablieren ein Medizinisches Datenintegrationszentrum (MeDIC), das auf offenen und international anerkannten Interoperabilitätsstandards basiert. Der Mehrwert der entwickelten Lösungen konnte durch klinische Anwendungsbeispiele in den Bereichen Kardiologie, Onkologie und Infektionskontrolle nachgewiesen werden. Durch die Schaffung von 11 neuen Professuren sowie 7 Nachwuchsgruppen wurde ein bedeutender Beitrag für die wissenschaftliche Lehre geleistet. Mit der Gründung des HiGHmed e. V. im Jahr 2019 sind zudem die notwendigen Grundlagen gelegt, um die entwickelten Lösungen langfristig zu etablieren und weiterzuentwickeln.

#### MIRACUM – Medical Informatics in Research and Care in University Medicine

Das MIRACUM-Konsortium [6] umfasst 10 DIZ-Standorte (Dresden, Erlangen, Frankfurt, Freiburg, Gießen, Greifswald, Magdeburg, Mainz, Mannheim, Marburg) und 2 Hochschulen (Mannheim, Mittelhessen). 2 nichtuniversitäre Krankenhäuser haben sich dem Konsortium 2023 als assoziierte Partner angeschlossen (Chemnitz, ZI Mannheim) und mit dem Aufbau eines DIZ begonnen. Der Mehrwert der in den DIZ etablierten Infrastrukturen wurde belegt anhand klinischer Use Cases (verteiltes maschinelles Lernen für COPD/Asthma [9–12], IT-Unterstützung für molekulare Tumorboards [13–18]) und eines methodischen Use Case (IT-Unterstützung bei der Patientenrekrutierung [19–22]). Zum Ausbau der medizinischen Informatik an den MIRACUM-Standorten wurden insgesamt 16 neue Professuren und 6 Nachwuchsforschergruppen eingerichtet. Die medizininformatische Aus- und Weiterbildung wurde durch Initiierung des berufsbegleitenden Studiengangs „Biomedizinische Informatik und Data Science“ an der Hochschule Mannheim gestärkt. Eine große konsortienübergreifende Resonanz fanden darüber hinaus die regelmäßige MIRACUM-Kolloquiumsreihe sowie die jährlichen Summer-Schools, die ab 2023 gemeinsam mit DIFUTURE veranstaltet wurden.

#### SMITH – Smart Medical Technology for Healthcare

In der Aufbau- und Vernetzungsphase der MII (2018–2022) konnte das SMITH-Netzwerk 7 DIZ an den universitätsmedizinischen Standorten Aachen, Bonn, Essen, Halle, Hamburg, Jena und Leipzig nachhaltig etablieren [7]. Die DIZ an den Universitätskliniken Düsseldorf und Rostock sowie die Ruhr-Universität Bochum befinden sich aktuell im Aufbau. Die erarbeiteten IT-Lösungen werden anhand klinischer und methodischer Anwendungsfälle erprobt und belegt: SMITH entwickelte in diesem Zusammenhang im klinischen Einsatz befindliche mobile Anwendungen auf dem Gebiet der Intensiv- und Infektionsmedizin (Use Case ASIC zur algorithmischen Überwachung in der Intensivversorgung, für Patientinnen und Patienten mit akutem Lungenversagen (ARDS); Use Case HELP zur App-basierten Unterstützung einer zielgerichteten Antibiotikatherapie) sowie innovative Verfahren, um aus elektronischen Patientenakten und Arztbriefen automatisiert medizinische Informationen gewinnen zu können (Use Case PheP). Der Förderung der medizininformatischen Lehre wurde durch die Einrichtung von 6 neuen Professuren, 5 Nachwuchsforschergruppen und 3 Studiengängen Rechnung getragen (siehe auch Beitrag von Knaup-Gregori et al. in diesem Themenheft).

### Weitere Fördermodule: Use Cases und Digitale FortschrittsHubs

Bereits in der Aufbau- und Vernetzungsphase wurden vom BMBF weitere Förderformate im Rahmen der MII etabliert: Die Förderung der 3 ersten *konsortienübergreifenden Use-Case-Projekte* von 2019 bis 2022 sollte die Vernetzung und die gemeinsame Datennutzung innerhalb der MII-Standorte fördern: Im Projekt POLAR (*Pol*ypharmazie, *A*rzneimittelwechselwirkungen und *R*isiken) wurde untersucht, inwieweit stationäre Versorgungsdaten zur Detektion von Medikationsrisiken bei Patientinnen und Patienten mit Polymedikation beitragen können [23]. Das Verbundprojekt CORD-MI (Collaboration on Rare Diseases) entwickelte Konzepte zur verbesserten einheitlichen Dokumentation von Seltenen Erkrankungen [24, 25]. Das Projekt ABIDE_MI (Aligning Biobanking and DIC Efficiently) erarbeitete schließlich Lösungen, damit die DIZ Patientendaten aus der Routineversorgung mit Daten zu Bioproben verknüpfen und für die Forschung nutzbar machen können [26]. Gleichzeitig legte es einen wichtigen Grundstein für das heutige deutsche Forschungsdatenportal für Gesundheit (FDPG; [27]).

Die 6 *digitalen FortschrittsHubs* (seit 2021) sollen insbesondere die regionale Vernetzung fördern und Gesundheitsdaten außerhalb der Universitätskliniken integrieren (siehe auch Beitrag von Krefting et al. in diesem Themenheft). Um die Medizininformatik in Deutschland zu stärken und dem Fachkräftemangel entgegenzuwirken, konnten sich Standorte, die *neue Professuren* im Bereich der Medizininformatik eingerichtet hatten, um die Einrichtung einer *Nachwuchsgruppe* bewerben.^1^

**Abb. 4:**
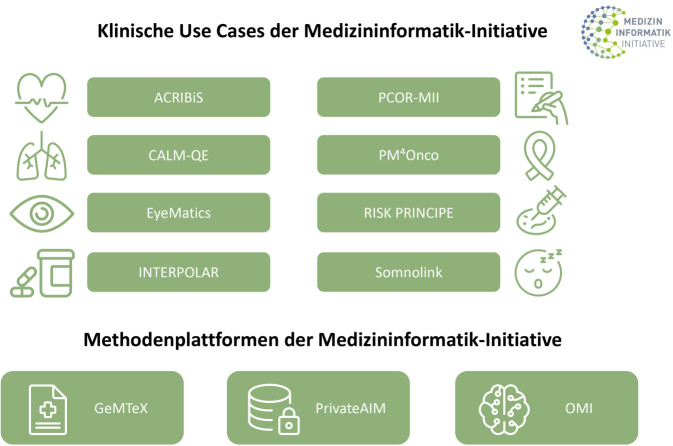
Klinische Use Cases und Methodenplattformen der Medizininformatik-Initiative (MII)

Seit dem Beginn der Ausbau- und Erweiterungsphase (2023) der MII sind weitere *übergreifende Use-Case- und Methodenplattform-Projekte* hinzugekommen, an denen sich obligat Standorte aller Konsortien beteiligen und die unterschiedliche Indikationsgebiete oder methodische Entwicklungen adressieren (Tab. 1 und Abb. 4). Diese sollen zur Erweiterung der Forschungsinfrastrukturen und deren Nutzbarkeit für unterschiedliche Bereiche der medizinischen Forschung beitragen (siehe auch die Beiträge von Metzger und Boerries, Loeffler et al. und Marschollek et al. in diesem Themenheft).

**Tab. 1:** Neue zentrale Infrastrukturprojekte und klinische sowie methodische Use Cases der Medizininformatik-Initiative (MII) ab 2023

Projektname	Inhalt	Projektstart
*Zentrale Infrastruktur- und Dienstleistungsprojekte*		
4C4MII (Coordination, Communication, Consultation, Convergence for the Medical Informatics Initiative)	Übergreifende Koordinierung aller zentralen MII-Aktivitäten und Aufbau/Weiterentwicklung/Betrieb des Deutschen Forschungsdatenportals für Gesundheit (FDPG; gemeinsam mit dem Projekt FDPG-PLUS)	01.07.2023
baseTRaCE (Basic services for Training and Continuous Education within the MII)	Koordination und Bündelung aller Fort- und Weiterbildungsmaßnahmen für die Standorte und Konsortien der MII	01.07.2023
DSF-Community	Standardisierte und sichere Kommunikation zwischen allen zentralen und dezentralen IT-Infrastrukturkomponenten der MII	01.01.2023
EVA4MII („EVAluation research based on data from routine clinical care 4 the MII“)	Methodische Unterstützung der Evaluationsforschung in der MII	01.04.2023
FDPG-PLUS (Erweiterung des MII-Forschungsdatenportals für Gesundheit)	Kontinuierliche Erweiterung des Deutschen Forschungsdatenportals für Gesundheit unter Berücksichtigung der verschiedenen Stakeholder-Anforderungen	01.01.2023
fit4translation („competence enhancement and support of the development of medical device software under the regulatory framework of MDR & IVDR in the academic environment“)	Untersuchung von Fragestellungen rund um die Entwicklung und Herstellung von Medical-Device-Software unter akademischen Rahmenbedingungen sowie Etablierung von Best Practices	01.07.2023
MII_NUM	Kooperation der MII mit dem Netzwerk Universitätsmedizin insbesondere bei gemeinsamen Schulungsmaßnahmen sowie gemeinsamer Nutzung und Weiterentwicklung der jeweiligen Infrastrukturen	01.07.2023
SU_TermServ	Bereitstellung FHIR-basierter terminologischer Dienste	01.01.2023
TRANSIT	Als Datenmanagementstelle vervollständigt TRANSIT den konsolidierten Data-Use-and-Access-Prozess und damit die Infrastruktur der MII	01.01.2023
*Konsortienübergreifende klinische Use Cases*		
ACRIBIS (Advancing Cardiovascular Risk Identification with Structured Clinical Documentation and Biosignal Derived Phenotype Synthesis)	Entwicklung von Risikomodellen für Herz-Kreislauf-Erkrankungen	01.04.2023
CALM-QE („COPD and asthma: longitudinal and cross-sectoral real-world data for machine learning application for quality improvement and knowledge“)	Optimierung der Behandlung von Patientinnen und Patienten mit Asthma und COPD durch KI-basierte multidimensionale Risikomodelle	01.05.2023
EyeMatics (Eye Diseases „Treated“ with Interoperabel Medical Informatics)	Bereitstellung einer Analyseplattform mit Daten verschiedener stationärer und ambulanter Versorgungssysteme zur Verbesserung der intravitrealen operativen Medikamentengabe bei Augenerkrankungen	01.03.2024
INTERPOLAR (INTERventional POLypharmacy—Drug interActions—Risks)	Automatisiertes Erkennen und Vorbeugen unerwünschter Arzneimittelwechselwirkungen	01.01.2023
PCOR-MII (Patient-Centered Outcomes Research within the Medical Informatics Initiative)	Integrierte Auswertung von digitalen Patient-reported-Outcome-Fragebögen gemeinsam mit Outcome-relevanten klinischen Parametern	01.03.2024
PM4Onco (Personalized Medicine for Oncology)	Etablierung einer Infrastruktur, um Daten aus klinischer und biomedizinischer Forschung zu integrieren und die personalisierte Medizin in der Krebsbehandlung zu optimieren	01.05.2023
RISK PRINCIPE (Risk Prediction for Risk-stratified Infection Control and Prevention)	Etablierung einer datengesteuerten, risikostratifizierten Infektionskontrolle in Krankenhäusern	01.07.2023
Somnolink („connected sleep data along the patient path for better care of Obstructive Sleep Apnea“)	Optimierung der patientenzentrierten Diagnose und Behandlung von Patientinnen und Patienten mit obstruktiver Schlafapnoe	01.03.2024
*Konsortienübergreifende methodische Use Cases*		
GeMTeX (German Medical Text Corpus)	Verfügbarmachung anonymisierter medizinischer Texte aus der Patientenversorgung für die Forschung	01.06.2023
OMI (Open Medical Inference)	Etablierung einer Plattform, die eine verteilte Nutzung von Anwendungen künstlicher Intelligenz ermöglicht	01.07.2023
PrivateAIM (Privacy-preserving AI in Medicine)	Etablierung einer föderierten Plattform für datenschutzgerechtes maschinelles Lernen und Datenanalytik in der MII	01.04.2023

### Kooperationen und Koordination: Die MII im nationalen und internationalen Kontext

Die MII verfolgt nicht das Ziel, eine einzelne Studie oder ein spezifisches Forschungsprojekt durchzuführen – die genannten Use-Case-Projekte dienen der exemplarischen Umsetzung und Testung –, sondern schafft infrastrukturelle Voraussetzungen für eine Vielzahl künftiger Forschungsvorhaben. Für die Sekundärnutzung von Versorgungsdaten liegen diese Voraussetzungen an der Schnittstelle zu Datenstrukturen und Dokumentationsprozessen der Krankenversorgung. NSG und MII-Koordinationsstelle haben daher für die MII frühzeitig ein aktives „Partnering“ und Kooperationen mit Akteuren der deutschen Gesundheitsversorgung initiiert (z. B. im Bereich der Datenstandardisierung, siehe hierzu weiter unten, sowie der Nutzung der elektronischen Patientenakte und der Telematikinfrastruktur), ebenso mit weiteren nationalen Forschungsinitiativen wie der ebenfalls öffentlich geförderten Nationalen Forschungsdateninfrastruktur (NFDI). Auch wurde der Arbeitskreis Medizinischer Ethik-Kommissionen (AKEK) als wichtiger Partner in die MII eingebunden.

Eine besondere Rolle nimmt die Kooperation mit dem 2020 in der COVID-19-Pandemie gegründeten *Netzwerk Universitätsmedizin (NUM)* ein: Frühzeitig brachte die MII die bereits aufgebauten Strukturen in das entstehende NUM ein, um Versorgungsdaten zu COVID-19-Fällen in einer Datenplattform (COVID-19 Data Exchange Platform [CODEX]) zusammenziehen und auswerten zu können [28]. Auch konnten in der durch die MII im Aufbau befindlichen dezentral föderierten Forschungsdateninfrastruktur für „Real-World“-Versorgungsdaten aus der deutschen Universitätsmedizin bereits während der Pandemie erste Analysen von Versorgungsgeschehen durchgeführt werden [29, 30]. Hierfür wurde der Förderrahmen seitens des BMBF angepasst: Die Aufbau- und Vernetzungsphase der MII wurde um ein Jahr verlängert und die Förderung des NUM sieht eine Integration der MII-Infrastrukturen vor. Insbesondere wird für die Erweiterung von Fragestellungen und Indikationsgebieten über COVID-19 hinaus die CODEX-Plattform zur Routinedaten-Plattform des NUM (NUM-RDP) weiterentwickelt. Die DIZ der Standorte werden in die NUM-Förderung eingebettet und als Infrastrukturen auch für Projekte im Rahmen des NUM genutzt (NUM-DIZ). Die Kooperation zwischen MII und NUM wird zudem in einem eigenen Fördermodul der Ausbau- und Erweiterungsphase gestärkt: Die im NUM vertretenen klinischen Fachkreise sollen hierüber stärker in die Datennutzung der Routinedaten einbezogen werden und MII und NUM haben gemeinsam eine *Koordinierungsgruppe Gesundheitsforschungsdateninfrastrukturen (GFDI)* initiiert, welche dem Austausch und der Abstimmung aller diesbezüglich relevanten Akteure in Forschung und Versorgung dient. Über das NUM bietet sich insbesondere die Chance auf Verstetigung essentieller Infrastrukturen mit Bundesmitteln [31].

Auch die internationale Anschlussfähigkeit der MII wird verfolgt. Unter anderem gibt es Verzahnungen mit internationalen Initiativen wie der FAIR-Data-Initiative der Research Data Alliance und der Initiative „Observational Health Data Sciences and Informatics“ (OHDSI) zur Durchführung internationaler Studien basierend auf dem OMOP Common Data Model (siehe auch Beitrag von Waltemath et al. in diesem Themenheft). 2023 organisierte die MII zudem einen internationalen Austausch mit vergleichbaren Initiativen in den europäischen Nachbarländern Niederlande (Health-RI) und Schweiz (SPHN) als Auftakt zu Kooperationen im europäischen Raum.

## Ergebnisse der MII

### Data-Sharing-Zielbild und Vertragsrahmen

**Abb. 5:**
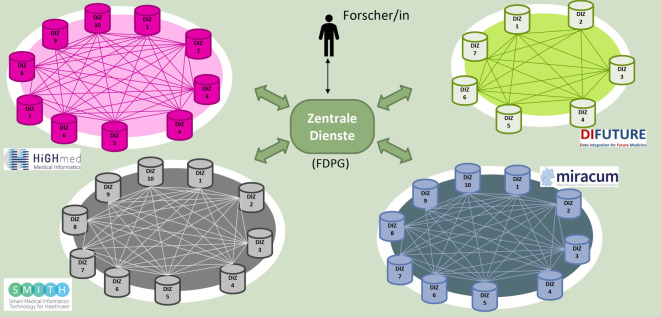
Data Sharing in der Medizininformatik-Initiative (MII) über die Datenintegrationszentren (DIZ) – am Standort, in den Konsortien und bundesweit übergreifend nach harmonisiertem Verfahren (über zentrale Dienste wie insbesondere das deutsche Forschungsdatenportal Gesundheit, FDPG)

Bereits in der Konzeptphase hat die MII ein durch ihre AG Data Sharing erarbeitetes Zielbild für das Teilen und die Nutzung von Daten formuliert: Eine dezentral föderierte Dateninfrastruktur soll harmonisiert an allen Universitätsmedizinstandorten und bei deren Partnern aufgebaut werden. Dies erlaubt eine verteilte Datenhaltung (im jeweiligen DIZ) an den Standorten, die die jeweiligen Patientinnen und Patienten behandeln und betreuen, und zugleich einen gemeinsamen Zugang zu diesen Daten nach einheitlichem Verfahren und in standardisierten Datenformaten. Diese harmonisierte Datennutzung erfolgt auf 3 Ebenen: am einzelnen Standort, im jeweiligen Konsortium und bundesweit übergreifend. Für den übergreifenden Zugang wird eine zentrale Antrags- und Zugangsstelle benötigt, das FDPG (Abb. 5).

Voraussetzung für die gemeinsame Nutzung von verteilt vorliegenden, in der Hoheit der einzelnen behandelnden Standorte befindlichen Patientendaten und Bioproben sind verbindliche und rechtlich wirksame Regelungen. Diese wurden über die AG Data Sharing etabliert in Form einer einheitlichen Nutzungsordnung, eines standardisierten Datennutzungsantrags und einer einheitlichen Nutzungsvertragsvorlage für die Datennutzungsverträge zu den einzelnen Forschungsvorhaben. Diese Regelungen und die damit verbundene Daten-Governance sind deutschlandweit mit allen teilnehmenden Partnern abgestimmt worden, das entsprechende Vertragswerk ist von allen Universitätsmedizinstandorten gezeichnet und seit Inkrafttreten bereits erfolgreich fortgeschrieben worden.^2^

Aktuell monitort die AG Data Sharing die ersten Datennutzungsprojekte und die Antrags- und Freigabeprozesse, in Interaktion mit DIZ-Leitungen und den für die Prüfung der Anträge und Freigabe von Daten verantwortlichen Use & Access Committees (UAC) an den Standorten (siehe auch Beitrag von Kirsten et al. in diesem Themenheft).

### Kerndatensatz und Interoperabilität

**Abb. 6:**
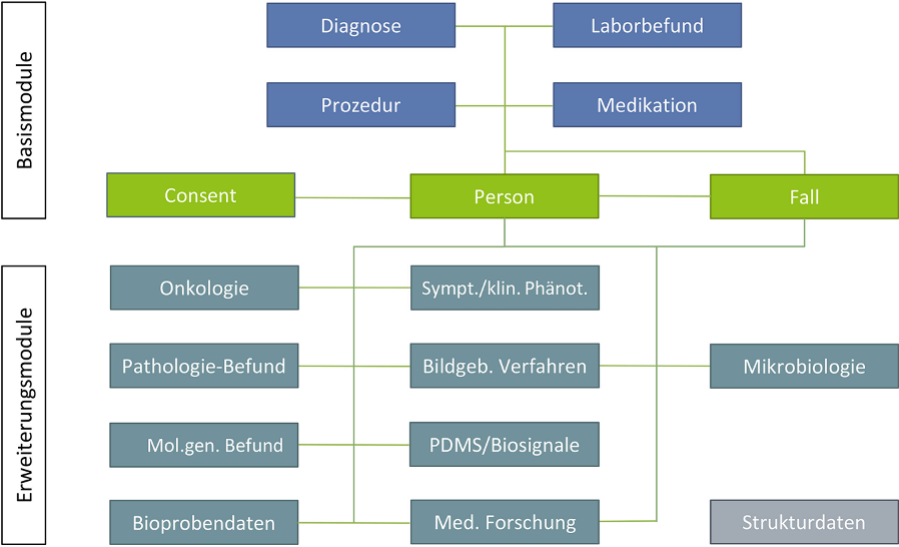
Kerndatensatz (KDS) der MII (Stand 2023). *klin. Phänot.* klinischer Phänotyp, *Med.* Medizinische, *Mol.gen.* molekulargenetischer, *PDMS* Patientendatenmanagementsysteme, *Sympt.* Symptome

Zentrale Voraussetzung für die gemeinsame Datennutzung ist die harmonisierte Datenausleitung: Definierte gleichartige Daten in einheitlichen Formaten müssen im Rahmen einer Forschungsanfrage von den Standorten bereitgestellt und für eine Analyse zusammengeführt und verteilt ausgewertet werden können. Auf Vorlage der AG Interoperabilität hat die MII festgelegt, dass ein *Kerndatensatz (KDS),* ein verbindlicher definierter Ausschnitt der Behandlungsdaten, aus den primären IT-Systemen der Patientenversorgung extrahiert und über das jeweilige DIZ in standardisierter Form verfügbar gemacht werden soll. Da die Daten in den primären IT-Systemen vielfach nicht standardisiert vorliegen, muss auch die Konvertierung in definierte Standardformate in diesem Zuge geleistet werden. Der MII-KDS ist modular aufgebaut und wird schrittweise definiert und implementiert: Prioritäre Basismodule (Daten zu Person, Fall, Consent, Diagnose, Prozedur, Laborbefund, Medikation) und sukzessive erarbeitete Erweiterungsmodule (Onkologiedatensatz, Pathologiebefund, molekulargenetischer Befund, Bioprobendaten u. a.) sind vorgesehen (Abb. 6) und werden in definierten Release-Plänen fortgeschrieben.^3^ In die inhaltliche Definition der Module werden die jeweiligen Fachgesellschaften und Fachgruppen einbezogen. Dabei ist die verbindliche Nutzung internationaler syntaktischer und semantischer Standards vorgeschrieben: Bereits 2017 wurde die Nutzung des Standards FHIR (Fast Healthcare Interoperability Resources) der Organisation HL7 (Health Level Seven International) festgelegt und dies in technischen Spezifikationen (sogenannte FHIR Implementation Guides) beschrieben. Ebenso wurde die verbindliche Nutzung von internationalen Nomenklaturen wie SNOMED CT (Systematized Nomenclature of Medicine – Clinical Terms) und (insbesondere für den Laborbereich) LOINC (Logical Observation Identifiers Names and Codes) festgelegt [32, 33]. Diese Festlegungen für den KDS der MII haben auch über den Rahmen der Gesundheitsforschung einen enormen Schub für die Nutzung von FHIR (und der hierfür erforderlichen Werkzeuge art-decor und Simplifier) und für die Anwendung von LOINC und SNOMED CT in Deutschland bedeutet [34]. Die MII arbeitet hierbei eng mit den Standardisierungsgremien zusammen, insbesondere werden die international gültigen Abstimmungsprozesse (Balloting) von HL7 Deutschland genutzt, um die Moduldefinitionen und FHIR-Spezifikation standardkonform umzusetzen und auch Akteuren jenseits der MII eine Mitwirkung an der Standardfestsetzung zu ermöglichen. Ebenfalls wurden frühzeitig eine Kooperation mit der Kassenärztlichen Bundesvereinigung (KBV) bzw. der KBV-Tochter mio42 GmbH und eine Entsendung von Expertinnen und Experten in die dortigen Gremien vereinbart, die sich mit der Festlegung der Inhalte der gesetzlichen elektronischen Patientenakte befassen. Hierdurch sollen Passfähigkeit von Standardfestsetzungen aufseiten von Forschung und Versorgung gesichert werden. SNOMED CT wurde über die MII 2020 in Deutschland mit einer deutschlandweiten Lizenz eingeführt (mit der Funktion des sogenannten National Release Center für SNOMED CT in Deutschland bei der TMF als Koordinationsstelle der MII); 2021 wurde die SNOMED-CT-Nutzung dann ausgeweitet auf den Bereich der Patientenversorgung und die Zuständigkeiten konnten in diesem Zuge auf das Bundesministerium für Gesundheit (BMG) und das Bundesinstitut für Arzneimittel und Medizinprodukte (BfArM) übergeben werden. Mit dem aktuell für die Interoperabilität im Gesundheitswesen zuständigen INTEROP Council bei der Gematik wie auch mit dem Beirat der Gematik gibt es personelle Verzahnungen der MII. Aktuelle Gesetzgebung sieht künftig eine weitere Stärkung der Interoperabilitätsbestrebungen vor, die AG Interoperabilität wird sich hierbei für die MII einbringen (siehe auch Beitrag von Ammon et al. in diesem Themenheft).

### Deutschlandweit harmonisierter Broad Consent als Rechtsgrundlage

Als Rechtsgrundlage für die Datennutzung von Patientendaten zu Forschungszwecken ist eine datenschutzkonforme informierte Einwilligung der Erkrankten erforderlich. Für eine spätere Nachnutzung von Daten aus der Patientenversorgung stellt sich hierbei die Herausforderung, dass für die Aufbereitung und Vorhaltung im DIZ bereits eine Einwilligung notwendig ist und dass die Patientinnen und Patienten zu einem Zeitpunkt um ihre Einwilligung befragt werden müssen, zu welchem die unterschiedlichen einzelnen späteren Forschungsfragestellungen, für die diese Daten in pseudonymisierter Form genutzt werden sollen, noch gar nicht bekannt sind. Die MII hat hierfür durch ihre Arbeitsgruppe Consent einen bundesweit einheitlichen Einwilligungstext für eine nach europäischer Datenschutzgrundverordnung grundsätzlich zulässige „breite Einwilligung“ (Broad Consent) zur Sekundärnutzung klinischer Daten entwickelt und mit allen Datenschutzaufsichtsbehörden der Länder und des Bundes sowie mit Vertretern der Ethikkommissionen abgestimmt. 2020 fand dieses Vorgehen die formale Genehmigung durch die Datenschutzkonferenz der Datenschutzaufsichtsbehörden.

Der von der MII entwickelte Broad Consent sieht mehrere Module vor, die separate Optionen der Einwilligung zur Nutzung z. B. von Bioproben oder Krankenkassendaten der betreffenden Person vorsehen [35]. Die Vorlage wird perspektivisch erweitert durch noch mit den Datenschutzaufsichtsbehörden und Ethikkommissionen abzustimmende weitere Module. Ergänzt werden die Aufklärungs- und Einwilligungsdokumente durch eine umfassende Handreichung zu dessen Einsatz an den Standorten sowie Hilfsmaterialien zur Informierung der Patientinnen und Patienten.^4^

Seit der erfolgten Genehmigung 2020 findet der Broad Consent sukzessive Anwendung an den Standorten der Universitätsmedizin. Auch die digitale Einholung der Einwilligungserklärung wird pilotiert [36]. Die AG Consent begleitet und berät diesen Prozess kontinuierlich und monitort die Verfahren zur Einholung der Einwilligungen. Insgesamt wurden bereits über 170.000 Einwilligungen zur grundsätzlichen Nachnutzung von Versorgungsdaten zu Forschungszwecken gemäß MII-Verfahrensweisen (Nutzungsordnung) von Patientinnen und Patienten erteilt (siehe auch Beitrag von Zenker in diesem Themenheft).

Im Zuge der Weiterentwicklung wird künftig auch der durch das Gesundheitsdatennutzungsgesetz (GDNG) veränderte Rechtsrahmen eingearbeitet.

### Verknüpfung mit anderen Datenbeständen zu Forschungszwecken

Über die Verfahren der MII wird ein wichtiger Zugang zu Versorgungsdaten zu Forschungszwecken geschaffen. Gleichwohl bildet der über die DIZ erreichbare Datenbestand nur einen Ausschnitt aus dem medizinischen Versorgungsgeschehen ab, sodass für viele Forschungsfragen eine Verknüpfung mit anderen verfügbaren Daten zum gleichen Behandlungsfall wichtig ist: Dies beginnt mit der Zusammenführbarkeit von Versorgungsdaten, sofern der Patient bzw. die Patientin mehrere Universitätskliniken aufgesucht hat, und setzt sich fort mit der Verknüpfung mit Daten bei Krankenversicherungen, (Krebs‑)Registern, Melderegistern und weiteren datenzentrierten Initiativen. Technische und rechtliche Fragen der Datenverknüpfung (Data Linkage) sind daher wichtiger konzeptioneller Arbeitsbestandteil in der MII [37, 38]. In einer gemeinsam mit dem NUM betriebenen AG Externe Daten werden aktuell für unterschiedliche Projekte und Studien Lösungen für eine Datenverknüpfung erarbeitet. Dabei liegt der Schwerpunkt – nicht zuletzt aufgrund der bereits vorliegenden großen Anzahl an Einwilligungen zur verknüpften Krankenkassendatennutzung – auf Leistungsdaten der gesetzlichen Krankenversicherung, die eine Längsschnittbetrachtung der Inanspruchnahme der Gesundheitsversorgung durch Patientinnen und Patienten (Patient Journey) in der ambulanten Versorgung (Verordnungsdaten, Prozeduren), der stationären Versorgung und der Rehabilitation ermöglichen. In der AG wird ein Architekturkonzept entwickelt, wie Kassendaten mit den Versorgungsdaten gemeinsam abgefragt und für Forschende bereitgestellt werden können. Dieses Konzept soll 2024 in erste Prototypen übersetzt werden. Einbezogen in diese Arbeiten werden neben MII und NUM auch Expertinnen und Experten aus kooperierenden AGs des Deutschen Netzwerks Versorgungsforschung (DNVF) und der Deutschen Gesellschaft für Epidemiologie (AGENS-Gruppe) sowie aus der Nationalen Forschungsdateninfrastruktur (NFDI4Health).

### Kommunikation und Patientenpartizipation

Die AG Kommunikation übernimmt gemeinsam mit der MII-Koordinationsstelle für die MII die wichtigen Aufgaben der Projektkommunikation und Veranstaltungsorganisation der jährlichen MII-Symposien. Die AG erstellt kontinuierlich Inhalte für die bestehenden MII-Kommunikationskanäle wie Webseiten^5^, Newsletter und Social-Media-Kanäle und schafft die Voraussetzungen für übergreifende Pressearbeit und Planung von Krisenkommunikation. Ein wichtiger Schwerpunkt ist die Patientenpartizipation: Früh wurden Patientenorganisationen und -selbsthilfegruppen einbezogen in die Arbeiten u. a. zu den Informationstexten und Hilfsmaterialien zum Broad Consent, zum Design des Forschungsdatenportals und der darin verankerten Transparenzfunktion mit Informationen zu Datennutzungsprojekten und zur spezifischen MII-Webseite für Patientinnen und Patienten^6^. Auch hat sich die MII mit einer eigenen repräsentativen Bevölkerungsumfrage an der Akzeptanzforschung beteiligt; eine hohe Zustimmung in der Bevölkerung für eine Nutzung von pseudonymisierten Behandlungsdaten zur Forschung durch Universitäten und behandelnde Ärztinnen und Ärzte konnte 2019, bereits vor der Pandemie, erhoben werden [39]. Die erfolgreiche Patientenpartizipation in der MII wird in der aktuellen Förderphase durch die Etablierung eines ständigen Patientenbeirats weiter gestärkt.

### Aufbau der Datenintegrationszentren (DIZ)

An allen Standorten der Universitätsmedizin sind Datenintegrationszentren (DIZ) aufgebaut worden, die die Aufgaben der Extraktion von Versorgungsdaten aus den primären IT-Systemen, die Annotation und Aufbereitung und die harmonisierte Datenausleitung mit Anbindung an das FDPG gewährleisten. Neu aufgebaute Universitätskliniken wie auch erste nichtuniversitäre Krankenhäuser sind mit gleichartigen DIZ hinzugekommen. Über diese Grundaufgaben hinaus stellen die DIZ einen wichtigen Kompetenzkern und Anlaufpunkt für vielfältige Projekte am Standort und in der Region dar (siehe auch Beitrag von Albashiti et al. in diesem Themenheft).

### Das Deutsche Forschungsdatenportal für Gesundheit (FDPG)

Das Deutsche Forschungsdatenportal für Gesundheit (FDPG) wurde im Oktober 2022 als zentrales Zugangsportal zu den Datenschätzen der Universitätskliniken vorgestellt und kann seit Mai 2023 auch von Forscherinnen und Forschern außerhalb der MII als Zugangsportal zu den (Versorgungs‑)Daten der Universitätskliniken genutzt werden.^7^ Damit ist das große Ziel der MII erreicht: ein deutschlandweites, föderiertes Netzwerk, welches den zentralen Zugang zu den in den elektronischen Krankenakten aller deutschen Universitätskliniken dokumentierten Daten ermöglicht und damit neue standortübergreifende datenbasierte Forschungsmöglichkeiten eröffnet. Aktuell haben sich 31 deutsche Universitätskliniken mit ihren lokalen DIZ an dieses Portal angeschlossen. Weitere 8 DIZ werden in den kommenden Jahren etabliert und zukünftig den Datenschatz bereichern.

Das FDPG besteht aus einem *Machbarkeitstool*, das auf Vorarbeiten aus dem oben dargestellten, mit dem NUM gemeinsam durchgeführten CODEX-Projekt aufsetzt [28], einem F*orschungsprojekt-Antragsmanagement-Modul* und einem *Transparenzregister*, welches gemäß den Vorgaben der Patienteninformation zum Broad Consent den Patientinnen und Patienten jederzeit einen aktuellen Überblick über die Datennutzungsprojekte der MII gibt. Ein solcher zentraler Zugangspunkt für Patientinnen und Patienten zu Informationen über Datennutzungen, noch vor Beginn derselben, wurde in den Abstimmungen mit den Datenschutzaufsichtsbehörden als wichtige kompensatorische Maßnahme für die Genehmigung des Broad Consent vorgesehen.

Die wichtigsten Funktionen des FDPG sind zum heutigen Zeitpunkt [40]:

- das Bereitstellen eines Überblicks über die in allen DIZ verfügbaren Daten und Datentypen,
- die Charakterisierung einer Kohorte für eine geplante Forschungsanalyse,
- das Durchführen von Machbarkeitsabfragen, um den Umfang der passend verfügbaren Patientendatensätze zu ermitteln,
- das Einreichen und Verwalten von Datennutzungsanträgen, um Daten von allen integrierten DIZ anzufordern und letztendlich den passenden Zugang zu den beantragten Daten für ihre Forschungsprojekte zu erhalten,
- das Anzeigen aller auf Basis des Broad Consent durchgeführten Datennutzungsprojekte.

Je nach datenschutzrechtlich gegebener Nutzungsgrundlage können in den Forschungsprojekten entweder verteilte Auswertungen durchgeführt werden (der Algorithmus „geht zu den Daten“, die Daten verlassen die Standorte nicht) oder aber zentrale Auswertungen (bei Vorliegen des Broad Consent als Patienteneinwilligung können die Daten zentral zusammengeführt und „zum Algorithmus gehen“). Eine sichere Kommunikationsschicht zur Verbindung der zentralen FDPG-Softwaremodule und der dezentral in den DIZ angebundenen FHIR-Server wird durch das Data Sharing Framework (DSF) bereitgestellt, welches sich transparent in die Gesamtarchitektur des FDPG integriert [41].

## Fazit und Ausblick

Mit dem Dekadenprojekt MII wurde erstmalig eine Fördermaßnahme durch das BMBF geschaffen, die gezielt die Gesamtheit aller Standorte der Universitätsmedizin in deutschlandweiter Kooperation vernetzt, um so einen einmaligen, umfassenden Forschungsraum für die Nutzung von Gesundheitsdaten zu schaffen. Dabei legt die MII erfolgreich Grundlagen zur Gesundheitsforschungsdateninfrastruktur in Deutschland, insbesondere zur Sekundärnutzung von Behandlungsdaten zu Forschungszwecken.

Aus Anlass der COVID-19-Pandemie wurde mit dem NUM eine ähnlich breite Kooperationsstruktur geschaffen, die es nun indikationsübergreifend mit den Strukturen und Prozessen der MII zu integrieren und zu verstetigen gilt. Diese bereits begonnene Integration muss konsequent fortgeführt und ein gemeinsames Zielbild für MII und NUM als deutschlandweiter Daten- und Studienraum erarbeitet werden. Dieses Zielbild muss zum einen die Gesamtheit der Universitätsmedizin in ihren Aufgaben in der Krankenversorgung, der Forschung und Lehre umfassen. Gleichermaßen muss dieses Zielbild auch die Erschließung von Daten und die Gewinnung von Studienpatientinnen und -patienten in weiteren Krankenhäusern, der ambulanten Versorgung, dem öffentlichen Gesundheitsdienst und selbsterfasste Daten sowie die Verknüpfung mit weiteren Datenbeständen umfassen. Mit dem kürzlich verabschiedeten Gesundheitsdatennutzungsgesetz (GDNG) und dem in Planung befindlichen Europäischen Gesundheitsdatenraum (European Health Data Space, EHDS) bietet sich nun erstmalig die Chance, die von der Bundesregierung verantworteten regulatorischen und infrastrukturellen Maßnahmen zur Nutzung von Gesundheitsdaten für die Forschung strategisch und operativ eng aufeinander abzustimmen [42]. Die von der MII aufgebauten Infrastrukturen – insbesondere die verteilten, harmonisierten DIZ und das zentrale FDPG als Zugangspunkt – können in Verbindung mit dem NUM wichtige Aufgaben in der kommenden Architektur zum Anschluss Deutschlands an den EHDS übernehmen. Damit ist die Grundlage geschaffen, in der patientenorientierten Gesundheitsfürsorge und -versorgung deutlich effektiver zu werden und Deutschland als Forschungsstandort im internationalen Vergleich in eine Führungsposition zu bringen.

## Footnotes

1 Insgesamt wurden bis 2023 im Rahmen der MII 51 Professuren und 21 Nachwuchsgruppen eingerichtet. Eine aktuelle Übersicht ist auf der Webseite der MII zu finden (unter https://www.medizininformatik-initiative.de/de/konsortien/medizininformatik-lehre-und-fortbildung/ bzw. https://www.medizininformatik-initiative.de/de/konsortien/nachwuchsgruppen-fuer-die-medizininformatik/). Zugegriffen 02.02.2024.

2 Die jeweils aktuellen Fassungen des Vertragswerks und der Nutzungsordnung sind auf der Website der Medizininformatik-Initiative zu finden (https://www.medizininformatik-initiative.de/). Zugegriffen 02.02.2024.

3 Die jeweils aktuellen Release-Stände sind über die Website der Medizininformatik-Initiative zu erreichen (https://www.medizininformatik-initiative.de/de/ueber-die-initiative/ergebnisse). Zugegriffen 02.02.2024.

4 Die jeweils aktuellen Fassungen der Aufklärungs- und Einwilligungsdokumente und Hilfsmaterialien sind auf der Webseite der Medizininformatik-Initiative zu finden (unter https://www.medizininformatik-initiative.de/de/mustertext-zur-patienteneinwilligung). Zugegriffen 02.02.2024.

5 https://www.medizininformatik-initiative.de/. Zugegriffen 02.02.2024.

6 https://www.vernetzen-forschen-heilen.de/. Zugegriffen 02.02.2024.

7 https://www.forschen-fuer-gesundheit.de/. Zugegriffen 02.02.2024.
